# Age-related differences in affective control and its association with mental health difficulties

**DOI:** 10.1017/S0954579419000099

**Published:** 2020-02

**Authors:** Susanne Schweizer, Jenna Parker, Jovita T. Leung, Cait Griffin, Sarah-Jayne Blakemore

**Affiliations:** 1 Institute of Cognitive Neuroscience, University College London, London, UK; 2 Medical Research Council Cognition and Brain Sciences Unit, University of Cambridge, Cambridge, UK

**Keywords:** adolescence, affective control, emotion regulation, executive function, mental health

## Abstract

Difficulties in regulating affect are core characteristics of a wide range of mental health conditions and are associated with deficits in cognitive control, particularly in affective contexts, affective control. The current study explored how affective control relates to mental health over the course of adolescence. We developed an Affective Control Task, which was administered to young adolescents (11–14 years; *n* = 29); mid-adolescents (15–18 years; *n* = 31), and adults (22–30 years; *n* = 31). The task required individuals to sort cards according to continuously changing rules: color, number, or item type. There was a neutral condition in which items were shapes, and an affective condition, in which items were emotional facial expressions. Better affective control was associated with fewer mental health difficulties (*p* < .001, *R*^2^ = .15). Affective control partially accounted for the association between age group and mental health problems, *z* = 2.61, *p* = .009, Akaike information criterion = 484, with the association being strongest in young adolescents, *r* (27) = −.44, *p* = .018. Affective control further accounted for variance in the association between self-reported (but not experimental) emotion regulation and mental health (*z* = −3.44, *p* < .001, Akaike information criterion = 440). Poor affective control, especially in young adolescents, is associated with more mental health problems and higher levels of emotion regulation difficulties. Improving affective control therefore may constitute a promising target for prevention.

Rates of common mental health disorders, in particular, affective and anxiety disorders, have increased over the past decade in young people worldwide (e.g., Mojtabai, Olfson, & Han, 2016), with the trend being strongest in young women (Bor, Dean, Najman, & Hayatbakhsh, 2014; NHS Digital, 2016). Advancing our mechanistic understanding of the shared antecedents of common mental health disorders is critical for improving detection and prevention of, and early intervention for, emerging mental health problems in young people (Hagan et al., 2015). Dysregulated affect is a core characteristic of common mental health disorders (Hofmann, Sawyer, Fang, & Asnaani, 2012) and is associated with deficits in cognitive control (Joormann & Tanovic, 2015), particularly in affective contexts, affective control (Schweizer et al., 2018). The current study sought to investigate how affective control capacity relates to mental health across adolescence.

Affective control constitutes all three proposed facets of cognitive control: updating, inhibition, and shifting (Miyake & Friedman, 2012), applied in affective contexts. Studies in adults have shown that deficits in updating affective content in working memory are associated with difficulties disengaging from negative perseverative thinking across mental health problems (e.g., affective disorders; Koster, De Lissnyder, Derakshan, & De Raedt, 2011). Poor inhibitory capacity of affective material is associated with attentional biases toward disorder-relevant information (e.g., threatening information in anxiety disorders) in adults (Bar-Haim, Lamy, Pergamin, Bakermans-Kranenburg, & van IJzendoorn, 2007). Deficits in the ability to shift prepotent maladaptive response patterns to flexibly select situationally appropriate emotion and self-regulatory strategies are related to both internalizing (e.g., affective disorders, anxiety disorders) and externalizing (e.g., addiction) mental health problems in adults (Aldao, Nolen-Hoeksema, & Schweizer, 2010; Bonanno & Burton, 2013). The adult literature thus suggests that deficits in all three facets of affective control are associated with mental health problems.

Less is known about how these different components of affective control develop during adolescence and how they may be related to mental health outcomes across age. Studies of “hot” executive functions (i.e., executive functions applied in motivationally or affectively salient situations) suggest that affective control may have a more protracted developmental time course than “cool” cognitive control abilities (e.g., Prencipe et al., 2011). For example, cool cognitive control measured with tasks such as the digit span (which measures updating) or the color Stroop (which measures inhibition) appears to mature earlier in adolescence than do hot executive functions (Peterson & Welsh, 2014). Hot executive functions are typically operationalized with tasks such as the Iowa Gambling task and the Delayed Gratification task (Peterson & Welsh, 2014; but see Aïte et al., 2018; Carlson et al., 2005). These tasks require decision making and inhibition in the context of motivationally salient rewards or losses (Welsh & Peterson, 2014).

Inferences from studies on varying developmental trajectories of cool cognitive control versus affective control, however, are complicated by the fact that any age-related effects could be accounted for by task-specific differences between the hot and cool paradigms. Studies that longitudinally investigate the development of affective control are largely lacking (but see: Adolescent Brain and Cognitive Development study; Volkow et al., 2018). Cross-sectional studies that compare cool cognition using the same task show that affective relative to neutral inhibitory control is lower in adolescents compared with adults (Aïte et al., 2018; Schel & Crone, 2013; Somerville, Hare, & Casey, 2011). In contrast, affective compared with neutral updating capacity may remain stable from adolescence to adulthood (Cromheeke & Mueller, 2016; Mueller, Cromheeke, Siugzdaite, & Boehler, 2017).

A growing body of work is suggesting that affective control is related to adolescent mental health. Specifically, adolescents with mental health problems including depression, dysphoria, bipolar disorder, and anxiety (Bertocci et al., 2012; Ladouceur et al., 2009, 2013; Tavitian et al., 2014; Wante, Braet, & Mueller, 2018; Wante, Mueller, Cromheeke, & Braet, 2018) show altered performance, compared with healthy adolescents, on tasks that require the updating of affective information in working memory. Poor inhibitory control of affective stimuli and shifting between affective and neutral task demands have also been associated with more depressive (e.g., Davidovich et al., 2016; Lo & Allen, 2011; Maalouf et al., 2012; Wante, Mueller, Demeyer, Naets, & Braet, 2017) and anxiety (Waters & Valvoi, 2009) symptoms in adolescents (but see Kyte, Goodyer, & Sahakian, 2005). Because these studies included adolescent samples only, it remains unclear how the association between affective control and mental health develops between adolescence and adulthood.

To assess affective control and its association with mental health in adolescents and adults, we developed a novel task that was a modified, affective version of the Madrid Card Sorting Test (Barceló, 2003). This task requires individuals to sort cards according to continuously changing rules. Specifically, cards are sorted according to color, number of items, or item type. Items are either shapes in the neutral condition or emotional facial expressions in the affective condition. Successful performance (sorting cards with as few errors as possible) requires all facets of affective control: inhibition of task-irrelevant affective information, updating the content of working memory to engage and disengage with affective information depending on changing rules, and shifting between affective and neutral responses strategies. We chose social stimuli (faces) to introduce affective content in the task because of the relative salience of social context in adolescence (Schriber & Guyer, 2016).

To test whether good affective control is associated with better regulation of affective responses to social rejection, we modified the standard emotion regulation paradigm (e.g., McRae et al., 2012) to include images of social rejection (Elliott et al., 2012; for sample see Figure 1B) and neutral social scenes. Specifically, the task assesses individuals’ capacity to effectively implement an adaptive emotion regulation strategy, reappraisal (Silvers & Guassi Moreira, 2019). We selected images of social exclusion because social stressors experienced during adolescence have been shown to be particularly detrimental to mental health (Fuhrmann, Knoll, & Blakemore, 2015; Romeo, 2017). Depression, for example, has been proposed to be associated with hypersensitivity to social rejection, a risk that may be heightened during adolescence (Allen & Badcock, 2003). In contrast, individuals with good affective control are likely to be able to regulate their affective responses to rejection, thereby decreasing the risk of aversive consequences to mental health.

**Figure 1.**
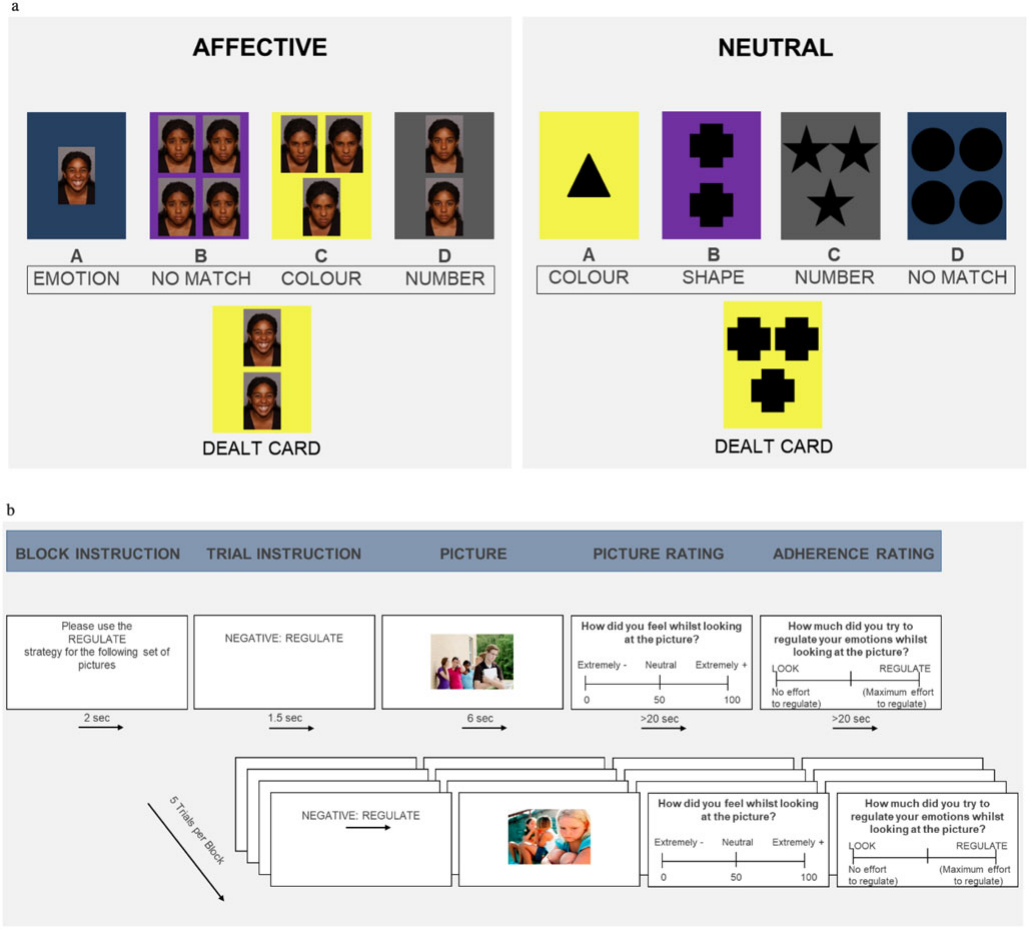
Sample trials of the Affective Control and Emotion Regulation tasks. (A) Affective Control Task showing sample trials in the affective (left) and neutral (right) conditions. Black box indicates the sorting rule for each of the four decks. (B) Emotion Regulation Task, which includes three different conditions: Regulate (example), Look Negative, and Look Neutral.

To down-regulate affective responses to scenes of social rejection, participants were asked to reappraise (i.e., reinterpret) the scene in a way that makes them feel less negative about the event. Poor capacity to implement adaptive regulation strategies such as reappraisal following negative events is prospectively associated with the experience of mental health problems, including depression (e.g., Kovacs, Rottenberg, & George, 2009). Reappraisal is a particularly well-researched, adaptive emotion regulation strategy (Buhle et al., 2014). Reappraisal has been shown to improve over the course of adolescence in experimental tasks (McRae et al., 2012; Silvers et al., 2012) and self-report studies on habitual use of cognitive complex reappraisal strategies (for a review, see Compas et al., 2017).

Difficulties in emotion regulation can extend beyond the implementation of situationally appropriate regulation strategies. Here, we used the self-report Difficulties in Emotion Regulation Scale (DERS; Gratz & Roemer, 2004) to assess other potential difficulties in the emotion regulation process including a lack of awareness of one's affective experiences, a lack of clarity about the type of affect experienced, and difficulties in overriding prepotent affective impulses in distressing situations.

The current study therefore investigated whether affective control is related to two aspects of emotion regulation: the capacity to implement adaptive emotion regulation strategies (i.e., reappraisal assessed on the Emotion Regulation Task) and difficulties with other aspects of the emotion regulation process (assessed with DERS; Gratz & Roemer, 2004). We further investigated whether good affective control partially accounts for the association between emotion regulation and mental health. Finally, we investigated whether these associations vary across adolescent development, by including participants from 11 to 30 years in our study.

The current study included only female participants to avoid the confound of age effects with differential timing of pubertal maturation in males and females (Patton & Viner, 2007). We were interested in the following hypotheses: first, we predicted that affective control, measured as the proportional difference in errors in the affective relative to neutral condition of the Affective Control Task, is associated with self-reported mental health problems (affective control and mental health hypothesis). Second, we predicted that this association varies as a function of age (developmental differences in affective control hypothesis). Third, we tested the hypothesis that good affective control is related to better emotion regulation (emotion regulation depends on affective control hypothesis). Fourth, we predicted that affective control partially accounts for the association between emotion regulation and mental health problems (affective control as buffer in negative contexts hypothesis). Finally, we explored whether the effect of affective control on the association between emotion regulation and mental health varies as a function of age.

## Method

### Participants

Ninety-one female participants were recruited from schools in and around London and Cambridge and departmental volunteer panels. Participants met the following inclusion criteria: no history of neurological disorders, head injuries, learning difficulties, or neurodevelopmental disorders. Informed consent was obtained from participants age 16 years and older and from caregivers for participants younger than age 18 years. Participants younger than age 16 years provided informed assent. The study was approved by the UCL Research Ethics Committee [Approval number: 9493/001] and the Cambridge Psychology Research Ethics Committee [Approval number: PRE.2016.053].

Participants were recruited into three a priori defined age groups of interest (Table 1): young adolescents (11–14 years), mid-adolescents (15–18 years), and adults (22–30 years). The age groups significantly differed in intelligence, as measured by the Matrix Reasoning subtest of the Wechsler Abbreviated Scale of Intelligence (Wechsler, 1999), *F* (2, 87) =7.36, *p* = .001, *R*^2^ = .14. The adult group scored significantly higher on the Matrix Reasoning subtest (Table 1) than both the early adolescent, *t* (87) = −2.96, *p* = .011, *d* = −0.77 and mid-adolescent, *t* (87) = −3.59, *p* = .002, *d* = −0.92, groups. There were no significant IQ differences between the two adolescent groups, *t* (87) < 1, *p* = .835, *d* = 0.15. Controlling for IQ in the analysis did not change the pattern of results and, because this reduced the sample size because of missing data, we report the results uncorrected for IQ in the main manuscript (see supplementary Materials for the results corrected for IQ).

**Table 1.** Participant characteristics

	Young adolescents *N* = 29	Mid-adolescents *N* = 31	Adults *N* = 31
Age *M* (*SD*)	13.51 (0.87)	16.53 (1.11)	25.9 (2.53)
Age range, years	11–14	15–18	22–30
IQ *M* (*SD*)	103.45 (13.63)	100.97 (16.90)	114.00 (10.70)

*Note:* IQ = Wechsler Abbreviated Scale of Intelligence Matrix Reasoning subtest (Wechsler, 1999). For a description of the ethnic distribution of the sample see supplementary Table S1.

### Tasks and measures

#### Affective control

The Affective Control Task (Figure 1A) was an affective version of the Madrid Card Sorting Test (Barceló, 2003); see Aker et al. (2014) for a similar task design based on the Wisconsin Card Sorting Test. Participants were dealt a card, which they assigned to one of four decks according to three possible sorting rules: card color, number of items and shape (neutral version), or emotional facial expression (affective version). Participants were instructed that the sorting rules would change randomly and to adopt a different sorting rule whenever they were informed that they had made an error. The rule switch occurred after nine trials. In this, the Affective Control Task deviated from the Madrid Card Sorting Test (Barceló, 2003), in which rule switches occur after six to eight trials. The rationale for increasing the block length to nine trials was that participants may take longer to establish sorting rules in the affective condition because it is harder to classify emotional expressions than more basic perceptual stimuli, which are used in the Madrid Card Sorting Test. Each sorting rule was presented twice in the neutral version (54 trials) and 4 times in the affective version (108 trials). Participants saw twice the number of affective trials to account for age-related variations in emotion recognition ability (Lawrence, Campbell, & Skuse, 2015). Trials were self-paced with a time limit of 1 min. That is, if no response occurred within 1 min, the trial was recorded as an error (in the current sample all responses were <1 min). The order of the affective and neutral versions was counterbalanced between participants.

Participants were exposed to all face stimuli during the task instruction phase. This was done to minimize differences in task performance resulting from the greater difficulty of detecting emotional expressions compared with shapes in the affective and neutral conditions, respectively. Specifically, participants saw four actors (two females) who displayed four different emotional expressions: happy, sad, fearful, and neutral.

Performance on the Affective Control Task can be operationalized as different types of error: nonefficient errors (failure to establish set, random and perseverative) as well as efficient errors (for details see the supplementary Materials). We had no a priori predictions regarding different interactions between task performance and affect across error types. Affective control was therefore operationalized as the proportional difference score between nonefficient errors in the number and color trials for the affective condition compared with the neutral condition (i.e., ((errors_affective_ – errors_neutral_) / errors_neutral_)). Proportional difference scores are commonly used in studies investigating age-related differences in affect-cognition interactions to account for potential age-related differences in overall error rates and/or variability (e.g., Lamm, Zelazo, & Lewis, 2006; Löckenhoff & Carstensen, 2007). We included performance on number and color trials only because they were not affected by the differential difficulty of the third sorting rule. Specifically, the third rule required emotional expression classification in the affective condition, which is perceptually more complex and demanding compared with classifying shapes (neutral condition). Moreover, because of the unequal number of trials between the affective (faces) and neutral (shapes) conditions, participants’ proportion errors were computed. These were then transformed (1 = proportion of errors) to enable the computation of the proportional difference score. The transformation was necessary because the affective–neutral difference could not be divided by zero in individuals who had no errors in the neutral condition. A higher score indicated fewer nonefficient errors in the affective relative to the neutral condition (i.e., better affective control).

The task had acceptable convergent validity, showing small to moderate associations with our measure of intelligence, the Matrix Reasoning task from the Wechsler Adult Intelligence Scale (Wechsler, 1999). The association between Matrix Reasoning and performance in the affective and neutral conditions, respectively, was *r* (87) = −.27, *p* = .009 and *r* (87) = −.22, *p* = .009. The task was programmed in E-Prime 2.0 (Schneider, Eschman, & Zuccolotto, 2002).

#### Emotion regulation

Emotion regulation capacity was assessed with an experimental Emotion Regulation Task (Figure 1B) and emotion regulation difficulties were assessed with a self-report measure (DERS; Gratz & Roemer, 2004). The DERS is a 36-item measure that provides an index of emotion regulation difficulties, with higher scores indexing greater emotion regulation problems (Gratz & Roemer, 2004). The scale has shown good psychometric properties in both adults (Gratz & Roemer, 2004) and adolescents (Weinberg & Klonsky, 2009). In the current sample, the reliability of the DERS was excellent, with Cronbach *α* = .92.

The Emotion Regulation Task used a standard picture-based task design (e.g., Silvers et al., 2012), in which participants were asked to look at negative and neutral images and either simply allow their emotions to arise (Look Negative and Look Neutral conditions) or to down-regulate their affective responses to the negative images (Regulate condition). The neutral images were neutral social scenes, whereas the negative images depicted scenes of social exclusion (for further details about the stimuli, see Elliott et al., 2012). After seeing each image, participants rated how they felt on a 7-point Likert scale, ranging from “very negative” to “very positive.” Following the affect rating, participants saw a second scale and were asked to indicate to what extent they had simply looked at the image or down-regulated their affective response.

Each of the 3 conditions was presented twice in blocks of 5 trials, with a total of 30 trials. Negative images were counterbalanced across the Look Negative and Regulate conditions. Block order was pseudo-randomized so that two identical blocks never followed each other and the task always ended with a neutral block.

Because the task including these images of social exclusion has not previously been used, we explored whether the conditions showed the expected patterns of affect ratings, that is, lowest mood rating in the Look Negative condition, intermediate levels in the Regulate Negative, and most positive mood in the Look Neutral condition. The effect of condition was significant, *F* (2, 172) = 226.60, *p* < .001, *R*^2^ = .56, and response patterns supported the task design. Reported mood was lower in the Look Negative (*M* = 3.82, *SD* = .93) compared with both the Regulate Negative (*M* = 4.27, *SD* = 1.21), *ΔM* = −0.45, *SD* = 0.95, *t* (86) = −4.45, *p* < .001, *d* = 0.42, and Look Neutral (*M* = 6.16, *SD* = 0.35) conditions, *ΔM* = −2.35, *SD* = 1.01, *t* (86) = −21.58, *p* < .001, *d* = 3.34. These two conditions in turn significantly differed in mood (*ΔM* = −1.90, *SD* = 1.29, *t* (86) = −13.76, *p* < .001, *d* = 2.13).

Emotion regulation capacity was computed by subtracting Look Negative ratings from the Regulate ratings. This index controls for variations in affective reactivity to negative information. The neutral condition was not further analyzed in the current study but was included for analysis in the larger study (see the following section). The task was programmed in E-Prime 2.0 (Schneider et al., 2002).

#### Mental health

Mental health problems were assessed with the Strengths and Difficulties Questionnaire (SDQ; Goodman, 1997) using the age-appropriate 11 to 17 years and the ≥18 years versions. The SDQ is a widely used screening tool for behavioral and emotional disorders in children and adolescents (Niclasen et al., 2012), with good convergent validity with clinician-rated diagnoses (He, Burstein, Schmitz, & Merikangas, 2013). The 25-item measure includes 5 subscales that assess internalizing (emotional problems subscale) and externalizing (hyperactivity and conduct subscales) symptoms, a subscale measuring difficulties with peers and a subscale assessing prosocial behavior. The 4 difficulties subscales (20 items) are summed to a total difficulty score, which has been shown to be indicative of youth mental health service use (Koskelainen, Sourander, & Kaljonen, 2000) and 12-month estimates of any *Diagnostic and statistical manual of mental disorders* (American Psychiatric Association, 2000) mental health disorder (He et al., 2013). In the current sample, the reliability of the items contributing to the difficulty score was acceptable Cronbach *α* = .68.

### Procedures

Participants were tested on a 14-inch laptop in quiet rooms either at their school, the UCL Institute of Cognitive Neuroscience, or the MRC Cognition and Brain Sciences Unit. The Affective Control Task was completed first, followed by two more cognitive tasks and questionnaires, which are part of a larger study and not described here. Participants then completed the experimental Emotion Regulation Task and finally the IQ measure and questionnaires. The total testing time was approximately 75 min. Participants received a £10 voucher for their participation.

### Statistical analyses

Participants with missing data on certain measures were excluded from the relevant analyses but retained for all other analyses to maximize power. Questionnaire data were missing from four participants (three adults and one mid-adolescent) and the experimental Emotion Regulation Task malfunctioned for four participants (two mid-adolescents and two adults). One adult participant did not complete the Affective Control Task faithfully by using only one response key for responses.

Before testing our hypotheses, we investigated the association between age group and mental health (SDQ) and emotion regulation capacity (experimental and self-report) using general linear modeling. We then tested the affective control and mental health hypothesis, which predicts a negative association between affective control and mental health problems. The hypothesis was tested with a linear regression analysis including mental health problems as the dependent variable and affective control (proportional difference score in nonefficient errors in the affective and neutral conditions; see the previous section) as the independent variable. To explore whether this association varies as a function of age group (developmental differences in affective control hypothesis), we next ran a mediation model. Mediation was selected not to imply longitudinal mediation, but to continuously model affective control rather than artificially segmenting it into dimensions to fit a standard moderation approach. The model included age group as the independent variable, affective control as mediator, and mental health problems as outcome.

We then tested our prediction that affective control (independent variable) is associated with behavioral and self-report measures of emotion regulation in two linear regression analyses (emotion regulation depends on affective control hypothesis). A mediation model with emotion regulation as the independent variable, affective control as mediator and mental health problems as outcome was run to test the affective control as buffer in negative social contexts hypothesis. We repeated this analysis with self-reported emotion regulation difficulties as the independent variable. Last, we explored the potentially moderating effect of age group on these mediation models. We ran a moderated mediation analysis including the same two mediation models with the addition of age group as moderator.

We also repeated the analyses with age as a continuous variable (although no data were collected between ages 19 and 21 years). The pattern of results remained unchanged (see supplementary Materials). In addition to our a priori hypotheses, we explored whether different types of error on the Affective Control Task show differential associations with age and mental health. For these exploratory analyses, we applied a Bonferroni correction to the significance threshold. That is, the comparison of the three error types (efficient, perseverative, and random) meant that results were considered significant at *p* ≤ .017. Finally, based on the literature on affective control in adults (Schweizer et al., 2018), we predicted that accuracy, but not reaction times (RTs), is associated with mental health. To evaluate this, we report all main analyses for RTs. Affective control RT was computed a proportional difference score analogous to the accuracy analyses:, (RT_affective_ – RT_neutral_) / RT_neutral_).

All analyses were run in R (R Core Team, 2013). Mediation models were analyzed using the lavaan package (Rosseel, 2012), with the exception of the moderated mediation analysis, which was computed using the PROCESS macro, version 3.0 (Hayes, 2013), in IBM SPSS, version 24 (IBM Corp., 2017). Alpha levels for all analyses were set at *p* = .05. Univariate comparisons deconstructing any significant effects were Tukey adjusted. Effect sizes are reported (*R*^2^ for all *F* tests and Cohen *d* for *t* tests). To calculate the effect sizes for paired-sample *t* test, an online effect size calculator was used (https://www.ai-therapy.com/psychology-statistics/). The full data analyses and figures script are available at https://osf.io/gwxhj/.

## Results

### Mental health, affective control, and emotion regulation capacity across age groups

#### Mental health

**Figure 2.**
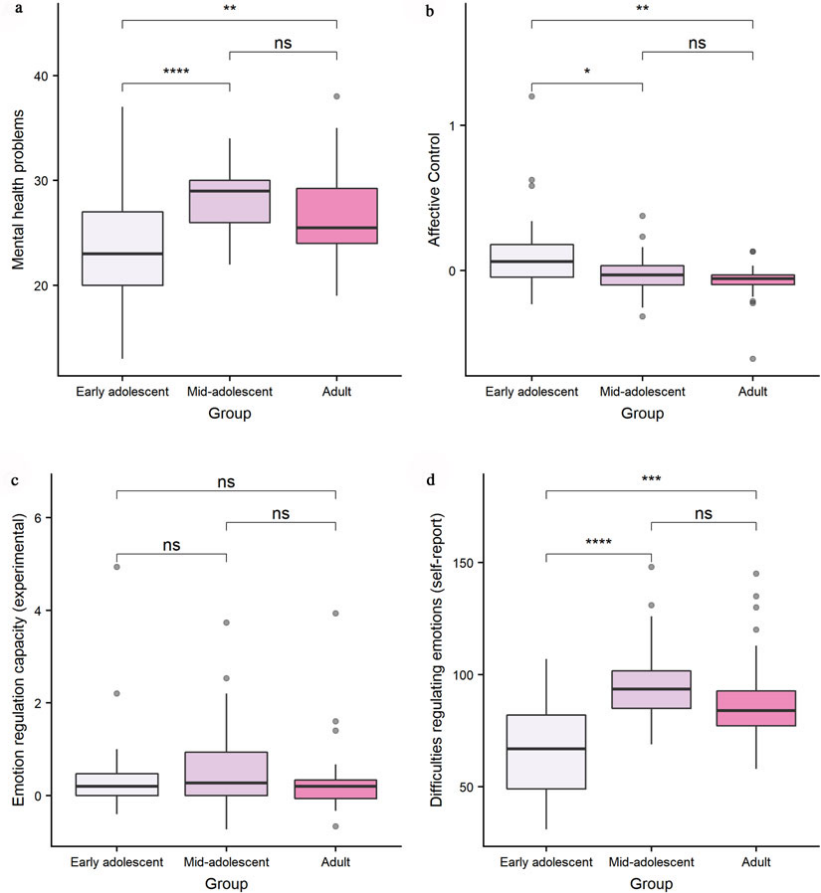
Association of age group with mental health, affective control, and emotion regulation. Bar graphs showing data from the early adolescent (11–14 years, *n* = 29), mid-adolescent (15–18 years, *n* = 31), and adult (22–30 years, *n* = 31) groups in each measure. (A) Mental health difficulties = SDQ total score (excluding the prosocial subscale), with higher scores indicating greater levels of mental health difficulties. (B) Affective control = proportional difference in performance in the affective condition relative to the neutral condition on the Affective Control Task, with higher scores indicating greater affective control. (C) Emotion regulation capacity (experimental) = the difference in distress ratings in the Regulation compared with Negative Look conditions of the Emotion Regulation Task. (D) Difficulties in regulating emotions (self-report) = DERS total score, with a higher score indicating more emotion regulation difficulties. ns = not significant; SDQ = Strengths and Difficulties Questionnaire. *p ≤ .05, **p ≤ .01, ***p ≤ .001, ****p ≤ .0001.

There was an effect of age group on mental health difficulties as measured by the SDQ: *F* (2, 84) = 11.46, *p* < .001, *R*^2^ = −.21 (Figure 2A). This was due to significantly fewer mental health difficulties in the early adolescent group compared with both the mid-adolescent, *t* (84) = −4.69, *p* < .001, *d* = −1.22 and adult, *t* (84) = −3.17, *p* = .006, *d* = −0.84 groups. There was no significant difference in self-reported mental health problems between the mid-adolescent and adult groups: *t* (84) = 1.45, *p* = .321, *d* = 0.38

.

#### Affective control

The groups differed significantly in affective control, *F* (2, 87) = 6.99 *p* = .002, *R*^2^ = .14 (Figure 2B). The early adolescent group showed significantly better affective control compared with both the mid-adolescent, *t* (87) = 2.68, *p* = .024, *d* = 0.69, and adult, *t* (87) = −3.61, *p* = .002, *d* = 0.94 groups. The latter two did not differ from each other in affective control capacity, *t* (87) <1, *p* = .595, *d* = 0.25. Alpha levels for the three univariate comparisons were Tukey adjusted. To explore whether the effect of age group was uniform in the neutral and affective conditions, we looked at the effect of age group for each condition separately. The effect of age group showed the same association pattern for both the affective, *F* (2, 87) = 1.87, *p* = .160, *R*^2^ = .04, and neutral, *F* (2, 87) = 17.98, *p* < .001, *R*^2^ = .29, conditions, but was significant only for the neutral condition.

Investigating affective control performance across the different types of error (Table S2) showed no effect of age group on the proportional difference of affective relative to neutral efficient errors, *F* (2, 87) <1, *p* = .949, *R*^2^ = .00. These were therefore not investigated further in any exploratory analyses. The overall effect of age group observed for nonefficient errors (main analysis) was due to significant age-related proportional differences in affective relative to neutral random errors, *F* (2, 87) = 7.56, *p* < .001, *R*^2^ = .15. There was no significant effect of age group on proportional differences in affective relative to neutral perseverative errors, *F* (2, 87) = 1.19, *p* = .309, *R*^2^ = .03. Again, the significant effect of age on random errors showed the same pattern for affective, *F* (2, 87) = 3.67, *p* = .030, *R*^2^ = .08, and neutral errors, *F* (2, 87) = 25.37, *p* < .001, *R*^2^ = .37, but was stronger in the neutral condition.

#### Emotion regulation

There was no effect of age group on the experimental Emotion Regulation Task, *F* (2, 84) <1, *p* = .589, *R*^2^ = .01 (Figure 2C). Self-reported emotion regulation difficulties, however, showed a significant age group effect (Figure 2D), *F* (2, 84) = 16.15, *p* < .001, *R*^2^ = .28, with young adolescents reporting significantly fewer emotion regulatory difficulties compared with both mid-adolescents, *t* (84) = −5.46, *p* < .001, *d* = −1.42, and adults, *t* (84) = −4.09, *p* < .001, *d* = −1.08. The mid-adolescent and adult groups did not significantly differ from each other, *t* (84) = 1.29, *p* = .405, *d* = 0.34.

Given the differential association between age group and the two measures of emotion regulation, we explored their association with each other. Performance on the Emotion Regulation Task was not significantly related to self-reported emotion regulation difficulties (Table S3), *r* (82) = .11, *p* = .316.

### Affective control and mental health

Supporting the affective control and mental health hypothesis, self-reported mental health difficulties on the SDQ, *F* (1, 84) = 15.28, *p* < .001, *R*^2^ = .15, were significantly associated with affective control performance. The association was negative, indicating that lower affective control was associated with more mental health difficulties, *r* (84) = −.39. Looking at random and perseverative errors separately showed a significant correlation with mental health problems at the Bonferroni corrected significance threshold of *p* ≤ .017 for random, *r* (84) = −.39, *F* (1, 84) = 15.30, *p* = .0002, but not perseverative, errors, *r* (84) = .21, *F* (1, 84) = 4.01, *p* = .049.

**Figure 3.**
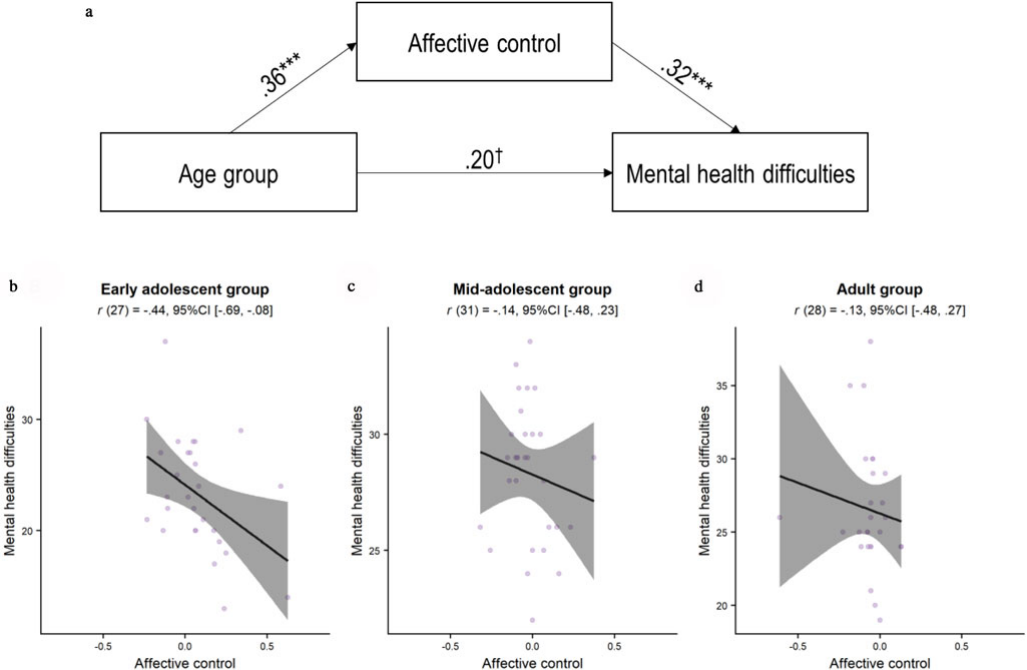
Differential association between affective control and mental health across age groups. (A) Significant indirect effect of affective control on the association between age group (i.e., young adolescent, mid-adolescent, adult) and self-reported mental health difficulties on the SDQ. The paths include *β* estimates of the associations. (B–D) Deconstruction of the indirect effect by showing the association between affective control and mental health in each age group separately. Mental health difficulties = SDQ total score (excluding the prosocial subscale), with high scores indicating greater levels of mental health difficulties; affective control = proportional difference in performance in the affective condition relative to the neutral condition on the Affective Control Task, with higher scores indicating greater affective control. SDQ = Strengths and Difficulties Questionnaire. ***p ≤ .001.

In line with the developmental differences in affective control hypothesis, affective control partially accounted for the association between age group and mental health problems; standardized indirect effect: *β* = 0.14, *SE* = 0.06, *z* = 2.61, *p* = .009, Akaike information criterion (AIC) = 483.58 (Figure 3A). To elucidate this effect further we looked at the association between affective control and mental health problems in each age group separately, showing a significant association in the early adolescent group, *F* (1, 27) = 6.36, *p* = .018, *R*^2^ = .19 (Figure 3B), but not in the mid-adolescent, *F* (1, 28) < 1; *p* = .451, *R*^2^ = .02 (Figure 3C) or adult groups, *F* (1, 25) < 1; *p* = .405, *R*^2^ = .02 (Figure 3D).

### Affective control and emotion regulation

The hypothesis that *emotion regulation depends on affective control* received only partial support (Figure S1). The experimental emotion regulation index showed no significant association with affective control, *F* (1, 84) < 1, *p* = .591, *R*^2^ = .00. Affective control was, however, significantly associated with individuals’ self-reported emotion regulation difficulties, *F* (1, 84) = 9.99, *p* = .002, *R*^2^ = .11. The association was negative *r* (84) = −.33, indicating that lower affective control is associated with more emotion regulation difficulties. Exploring the random error and perseverative error components of the affective control measure separately showed that the proportional difference in random errors, *r* (84) = −.33, *F* (1, 84) = 9.92, *p* = .002, *R*^2^ = .11, but not perseverative errors, *r* (84) = −.33, *F* (1, 84) = 1.83, *p* = .178, *R*^2^ = .02, was significantly related to emotion regulation difficulties, when adopting a significance threshold of *p* ≤ .017 for multiple comparisons in exploratory analyses.

**Figure 4.**
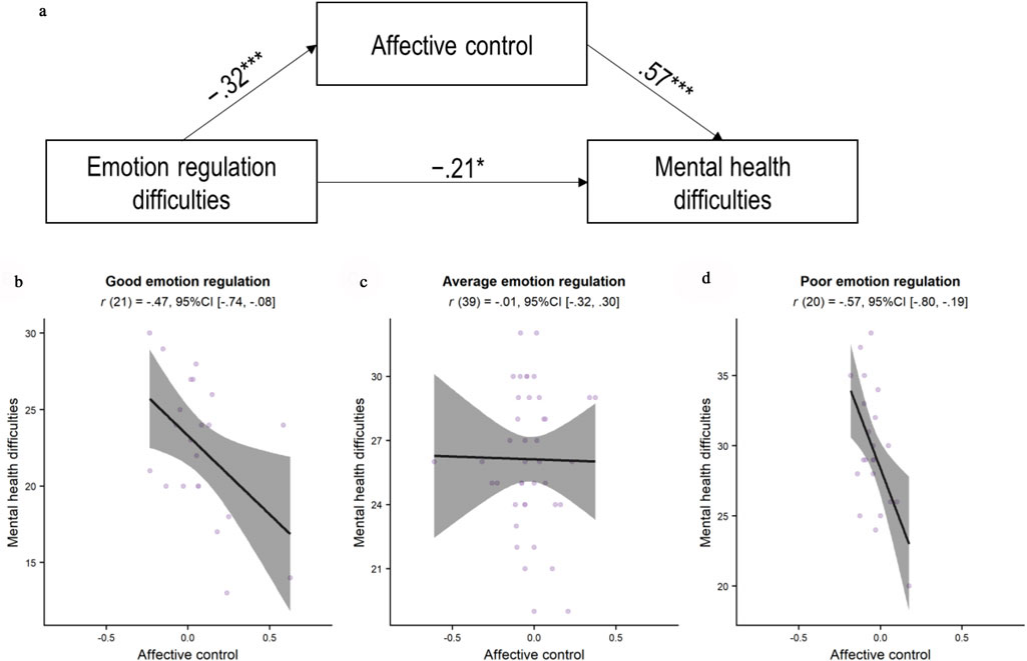
Differential association between affective control and mental health across emotion regulation capacity. (A) Significant indirect effect of affective control on the association between emotion regulation difficulties and mental health. The paths include *β* estimates of the associations. (B–D) Deconstruction of the indirect effect by showing the association between affective control and mental health across levels of emotion regulation capacity separately. Mental health difficulties = SDQ total score, in which high scores indicate greater levels of mental health difficulties (excluding the prosocial subscale). Affective control = proportional difference in performance in the affective condition relative to the neutral condition on the Affective Control Task, with higher scores indicating greater affective control. Good emotion regulation = DERS total score ≤72. Average emotion regulation = DERS total score of 73–96. Poor emotion regulation = DERS total score of *p* ≥ 97; *p ≤ .05, ***p ≤ .001.

The results supported the affective control as buffer for mental health in negative contexts hypothesis for self-reported emotion regulation difficulties. That is, participants’ affective control capacity partially accounted for the association between self-reported difficulties in emotion regulation and mental health difficulties, *β* = −0.87, *SE* = 0.25, *z* = −3.44, *p* = .001, AIC = 440.05 (Figure 4).

To explore this significant indirect effect, we divided the groups into individuals with low (≤first quantile, ≤72, *n* = 23), average (73–96, *n* = 42), and high (≥third quantile, ≥97, *n* = 22) levels of emotion regulation difficulties. Affective control differed significantly across the three groups, *F* (2, 83) = 4.93, *p* = .009, *R*^2^ = .11. Univariate analyses revealed that individuals who reported low levels of emotion regulation difficulties reported significantly greater affective control (*M* = .12, *SD* = .32) than both individuals reporting average, *t* (83) = 2.81, *p* = .017, *d* = 0.73, and high, *t* (83) = 2.73, *p* = .021, *d* = 0.81, levels of emotion regulation difficulties. There was no significant difference in affective control between individuals who reported high levels of emotion regulation difficulties (*M* = −.04, *SD* = .08), and those with average levels of emotion regulation difficulties (*M* = −.03, *SD* = .17), *t* (83) < 1, *p* = .948, *d* = 0.08. Affective control was significantly associated with mental health problems in both individuals reporting high, *F* (1, 20) = 9.58, *p* = .005, *R*^2^ = .32, and low *F* (1, 21) =6.09, *p* = .022, *R*^2^ = .22, levels of emotion regulation difficulties, but not in those with average levels, *F* (1, 39) < 1, *p* = .932, *R*^2^ = .00.

The mediation analysis for the experimental emotion regulation index was not meaningful, because emotion regulation was unrelated to mental health difficulties, *F* (1, 82) < 1, *p* = .89, *R*^2^ = .00, or age group and affective control (see the previous section). Moreover, our exploratory analysis showed no moderating effect of age group on the mediating effect of affective control on the association between self-reported emotion regulation capacity and mental health problems, index = −0.007, *SE*_*Bootstrap*_ = 0.009, 95% CI (−0.02, 0.01).

### Reaction time

For RT on the Affective Control Task, there was a significant effect of age group, *F* (2, 87) = 4.14 *p* = .019, *R*^2^ = .09 (see Table S2 for the RTs in each condition across the groups). The adult compared with the early adolescent group was significantly slower in the affective condition relative to the neutral condition, *t* (87) = −2.61, *p* = .028. Adults also showed greater slowing in the affective condition compared with the neutral condition than the mid-adolescent group, but this difference was not statistically significant, *t* (87) = −2.35, *p* = .054. There was no significant difference in RT between the two adolescent groups, *t* (87) < 1, *p* = .951.

There was no association between mental health and the proportional difference in RT for the affective condition relative to the neutral condition (affective control and mental health hypothesis), *F* (1, 84) < 1, *p* = .626, *R*^2^ = .00, nor did this association vary as a function of age group (developmental differences in affective control hypothesis), *β* = −0.005, *SE* = 0.03, *z* = −0.18, *p* = .861, AIC = 498.70. Affective control RT was not related to either self-reported emotion regulation, *F* (1, 84) < 1, *p* = .348, *R*^2^ = .01, or experimentally assessed emotion regulation, *F* (1, 84) < 1, *p* = .906, *R*^2^ = .00 (emotion regulation depends on affective control hypothesis). Affective control RT did not account for any variation in the association between emotion regulatory capacity and self-reported mental health problems (affective control as buffer in negative contexts hypothesis), *β* = 0.25, *SE* = 0.20, *z* = 1.21, *p* = .227, AIC = 454.50.

## Discussion

The current findings support the role of affective control in common mental health problems for both adolescents and adults. Performance on our Affective Control Task showed a moderate (*r* = −.43) association with mental health problems across age groups. In support of the developmental differences in affective control hypothesis, affective control also significantly accounted for part of the variance in the association between age group and mental health problems. Specifically, affective control was most strongly associated with mental health problems in early adolescence (11–14 years). The hypothesis that the effect of affective control on mental health is due to its role in emotion regulation was supported for self-reported emotion regulation difficulties, but not for emotion regulation as measured by an experimental Emotion Regulation Task. Together, these results suggest that affective control plays an important role in mental health, especially in early adolescence.

Young adolescents with the highest levels of mental health problems showed the poorest affective control performance. This is in line with previous research showing that, in young adolescents, cognitive flexibility in an affective context was significantly associated with symptoms of anxiety (Mărcuş, Stanciu, MacLeod, Liebregts, & Visu-Petra, 2016). Findings in adults have been more mixed. Although one study in adults with depression showed impaired performance on negative compared with neutral trials of an affective Wisconsin Card Sorting Test (Deveney & Deldin, 2006), a second study in adult women showed no association between impairments on an emotional card sorting task, similar to the Affective Control Task used in the present study, and depression (Aker et al., 2014). These findings, in combination with the current study, suggest that the association between affective control and mental health may not be uniform across development.

Applying cognitive control in affectively charged environments might be particularly challenging for young adolescents with vulnerabilities to mental health problems. Preliminary evidence for this suggestion stems from Davidovich et al. (2016), who showed that good affective inhibitory control buffered adolescents’ mental health against the risk factor of having a parent with depression. Affective control in young adolescents, especially those at risk for mental health problems, may therefore constitute a promising target for prevention interventions.

A known vulnerability factor for future mental health problems is poor emotion regulation capacity (Compas et al., 2017), which has been proposed to depend on the application of cognitive control in affective contexts (Ochsner & Gross, 2005). In contrast with our prediction, our experimental measure of emotion regulation capacity showed no significant association with mental health problems in any age group. The nonsignificant association between an experimental measure of emotion regulation and mental health outcomes has previously been shown in adolescents with depression and anxiety (e.g., Belden, Pagliaccio, Murphy, Luby, & Barch, 2015; Carthy, Horesh, Apter, Edge, & Gross, 2010). The lack of association observed in the current study may be partially accounted for by the fact that the Emotion Regulation Task measured the capacity to implement the use of reappraisal when instructed to do so in a laboratory context. This may not be a reliable indicator of an individual's capacity to flexibly select and implement adaptive regulation strategies in a real-world setting. In support of this argument, there was no association between Emotion Regulation Task performance and self-reported emotion regulation difficulties. In addition, the Emotion Regulation Task required participants to capture the affective narrative of complex social scenes and simultaneously regulate their affect in response to these scenes. These images of social rejection require significant cognitive effort to construct an affective response (Barrett, 2013). This effort is in contrast with the more visceral and immediate affective responses (Ewbank, Barnard, Croucher, Ramponi, & Calder, 2009) elicited by international affective picture system images (Lang, Bradley, & Cuthbert, 2008), which are commonly used in emotion regulation paradigms (Belden et al., 2015; Perlman et al., 2012). Although these interpretations remain speculative, the current results suggest that experimental measures of emotion regulation capacity in response to negative pictures do not constitute a sensitive marker of variability in adolescent mental health problems.

In contrast to performance on the Emotion Regulation Task, *self-reported* emotion regulation difficulties, ranging from lack of emotional awareness to difficulties in generating adaptive regulation strategies when distressed, showed a significant association with mental health problems. This association was partially accounted for by affective control. These findings are in line with previous work showing that students’ (18–23 years old) ability to successfully update affective content in working memory predicted their subsequent ability to regulate their emotions during a stressful examination period. Moreover, affective control capacity longitudinally mediated the association between stress exposure and subsequent symptoms of depression (Quinn & Joormann, 2015); however, this study did not compare the differential predictive utility of affective versus cool cognitive control. Recent work by Stange et al. (2016) showed that a measure of cool cognitive control, in particular the ability to flexibly shift attention between mental sets, predicted the onset of major depressive disorder across a 4-year period in adolescents aged 12 to 13 years at baseline. Thus, although the current study suggests that it is the difference between affective and neutral control capacity that may be most predictive, future research should test this hypothesis prospectively.

The role of affective control in emotion regulation may be particularly relevant in adolescence when emotion regulation demands are high (Riediger & Klipker, 2015), with adolescents experiencing more negative affect and more rapid fluctuations in affect (Larson, Moneta, Richards, & Wilson, 2002). In support of this proposal, we found that individuals who reported high levels of emotion regulation difficulties showed the strongest association between affective control and mental health. This suggests that affective control can buffer the association between situationally high-emotion regulatory demands and mental health problems. Although our results are cross-sectional and therefore preclude temporal inferences, they do provide preliminary evidence that tasks investigating the relative contribution of affective and neutral control may be more sensitive to variations in emotion regulation.

Of interesting, however, individuals who reported few emotion regulation difficulties also demonstrated a moderate association between affective control and mental health problems, whereas those with average levels of emotion regulation difficulties did not. The association between affective control and mental health in individuals reporting low levels of emotion regulation difficulties may be indicative of a role of affective control in processes other than affect regulation; for example, affective control may be related to better social functioning. Affective control, measured in children and young adolescents on an emotional Stroop task, was associated with peer victimization (Rosen, Milich, & Harris, 2007) and social rejection sensitivity (Martin & Cole, 2000). Social functioning in turn is associated with mental health outcomes (e.g., depression; Kupferberg, Bicks, & Hasler, 2016; Verboom, Sijtsema, Verhulst, Penninx, & Ormel, 2014). This is only one of many potential pathways, beyond emotion regulation, through which affective control might influence mental health outcomes, and these should be explored in future research.

The observed effect of affective control on the association between self-reported emotion regulation and mental health did not vary as a function of age in the current study. This exploratory analysis, however, needs to be interpreted with caution as it had limited power to detect a moderating effect of age group, because of the relatively small number of participants in each age group. This null finding would therefore need to be replicated in a larger sample.

One unexpected finding in the current study was that affective control was higher in young adolescents compared with the mid-adolescent and adult groups. This differential effect was found despite young adolescents making significantly more errors overall and being slower to perform the task irrespective of condition valence or error type. As noted previously, hot executive functions have typically shown age-related improvements. Measures that directly compare facets of affective control on tasks including neutral and affective conditions, however, have shown more mixed results. Performance on measures of pure inhibitory capacity of attention or responses toward affective relative to neutral information typically shows an improvement from early adolescence to adulthood (e.g., Aïte et al., 2018; Schel & Crone, 2013; Somerville et al., 2011), although not uniformly (Lamm et al., 2006). Affective updating, in contrast, may be fully developed by early adolescence. Cromheeke and Mueller (2016) and Mueller et al. (2017) found no differences in the capacity to accurately update happy, angry, or neutral faces on a two-back task in adolescents compared with in adults. RT for happy faces, however, was slowed in adolescents, but not in adults, when participants had to maintain the gender (compared with affective expression) of the face in working memory (Cromheeke & Mueller, 2016). Affective shifting ability, to our knowledge, has not been compared directly in adolescents and adults.

Our results should be interpreted in the context of the study's limitations. First, the measure of mental health was a self-report measure, which, although it has good convergent validity with clinical assessments of mental health problems (He et al., 2013), is not a diagnostic instrument. Another consideration is that any age-related effects may be partially accounted for by greater sensitivity to demand effects in younger compared with older participants. The study included females only, and affective control processes may show different associations with mental health in males. A further limitation is that, although the Affective Control Task requires participants to engage all three facets of affective control (i.e., inhibition, updating, and shifting), potentially different developmental trajectories on these components cannot be disentangled using this task. Future research should investigate these facets individually within a single sample to explore their shared and unique contributions to mental health outcomes.

A consideration with regard to the findings of the association between mental health and emotion regulation is that affective control was modeled as the mediator. This was done to investigate whether individuals with higher levels of affective control report fewer mental health problems because they possess the necessary cognitive resources to implement a situationally appropriate regulatory strategy (Selection, Optimization, and Compensation model of emotion regulation; Opitz, Gross, & Urry, 2012). Alternatively, affective control could have been modeled as the predictor, as affective control may contribute to the development of greater habitual use of adaptive emotion regulation strategies. However, investigating this relationship requires a longitudinal study design. A prospective study would also allow us to explore the interaction of affective control with other factors that influence habitual emotion regulation (e.g., early adversity [McCrory, De Brito, & Viding, 2011], intergenerational transmission [Buckholdt, Parra, & Jobe-Shields, 2014, and socialization [Thompson & Meyer, 2007]). More generally, the cross-sectional nature of the current study means that we can only draw inferences regarding the age-related variation in task performance. Elucidating the predictive utility of affective control in relation to the development of mental health problems across age is an important next step.

In conclusion, this study shows that poor affective control is associated with more mental health problems, especially in early adolescence. Affective control capacity mediated the association between self-reported emotion regulation difficulties and mental health problems, suggesting that affective control is indeed one of the cognitive building blocks of adaptive emotion regulation. These findings are promising in the context of previous work in adolescents and adults showing that affective control can be trained (e.g., Schweizer et al., 2017; Schweizer, Grahn, Hampshire, Mobbs, & Dalgleish, 2013). Future research should investigate the predictive validity of our new measure of affective control for mental health outcomes across adolescence.

## Supplementary material

For supplementary material accompanying this paper visit http://dx.doi.org/10.1017/S0954579419000099. click here to view supplementary material
