# Supplementary material for: Age-related differences in affective control and its association with mental health difficulties
Source: Dev Psychopathol. 2019 Mar 25;32(1):329–41. doi: 10.1017/S0954579419000099 (PMC6982534; doi:10.1017/S0954579419000099)
Supplement: Supplementary file 1 [file S0954579419000099sup001.pdf]

Supplementary Materials

Age-Related Differences in Affective Control and its Association with Mental Health Difficulties

Supplementary Methods

Error types on the Affective Control Task

Three-dimensional errors can occur on the initial trial when any rule is an option, as well as after shift trials. In these cases, the sorting rule is not knowable and three-dimensional errors are considered efficient errors. Two-dimensional errors can occur on the trial following a rule switch when participants have a 50% chance of choosing the correct one of two alternatives. Two dimensional errors can either be efficient errors or perseverative errors (Barceló & Knight, 2002). The former is committed to establish the new sorting rule. Perseverative errors occur when participants persist in using the previous sorting rule despite the feedback that they should switch rules. Following the second trial in a series, participants can make three different types of error: i) perseverative errors; ii) non-perseverative failures to establish set; or iii) failures to maintain set (Barceló, 1999; Barceló & Knight, 2002). These different types of error are the consequences of, respectively: i) a failure to break with an invalid sorting rule; ii) a failure to maintain in working memory the discarded sorting rules (i.e., switching between invalid rules); and iii) an apparent random lapse in attention after a sorting rule is established. In the Madrid Card Sorting Test, the latter two types of error are categorised as random errors (Barceló & Knight, 2002).

Descriptive data

Table S1. Participant self-identified ethnicity

	Young adolescents	Mid-adolescents	Adults
White	22	20	7
Asian	1	2	17
Black/African/Caribbean	0	4	1
Mixed-race	2	3	1
Prefer not to say	5	2	5

Table S2. Mental health and affective control across groups

	Young adolescents	Mid-adolescents	Adults
	N = 29	*N* = 31	*N* = 30
	M (*sd*)	*M* (*sd*)	*M* (*sd*)
**Mental health measures**			
Mental health difficulties (SDQ)*	23.14 (5.17)	28.33 (2.93)	26.71 (4.4)
Emotion regulation difficulties (DERS)*	67.55 (21.16)	96.17 (18.32)	89.36 (20.85)
**Affective control**			
Proportion non-efficient errors affective condition	.19 (.11)	.14 (.07)	.14 (.13)
Proportion non-efficient errors neutral condition	.24 (.15)	.11 (.10)	.07 (.08)
Proportional difference score non-effective errors†	.11 (.29)	–.03 (.14)	–.08 (.13)
Effective errors affective condition	.66 (.13)	.69 (.11)	.66 (.13)
Effective errors neutral condition	.62 (.18)	.63 (.13)	.61 (.17)
Different types of non-effective errors			

Perseverative errors affective condition	.04 (.03)	.03 (.03)	.04 (.08)
Perseverative errors neutral condition	.05 (.06)	.02 (.03)	.02 (.04)
Random errors affective condition	.15 (.09)	.11 (.05)	.10 (.06)
Random errors neutral condition	.19 (.11)	.09 (.08)	.05 (.05)
Reaction time			
Reaction time affective condition	1541.52 (616.10)	1348.73 (438.21)	1333.85 (890.66)
Reaction time neutral condition	1601.91 (504.14)	1401.31 (465.93)	1143.10 (407.40)
Proportional difference score reaction time	–.03 (.22)	–.01 (.25)	–.15 (.30)

Table S2. * mid-adolescent group: n = 30, adult group: n = 28; † please note the proportional difference score was computed with the transformed error score (see results).

Analyses including IQ as covariate

Mental health, affective control and emotion regulation capacity across age groups

The effect of age group on mental health problems, F (3, 82) = 7.56, $p \leq .001$, R^2 = .22, affective control, F (3, 85) = 4.56, p = .005, R^2 = .14, and self-reported emotion regulation difficulties, F (3, 82) = 10.64, $p \leq .001$, R^2 = .28 remained significant with the inclusion of IQ as a covariate. The effect of age group on the experimental measure of emotion regulation remained non-significant F (3, 82) < 1, p = .672, R^2 = .02 after the inclusion of IQ as covariate.

Affective control and mental health across age groups

The indirect effect of affective control on the association between age group and mental health problems remained significant after correcting for IQ, standardized indirect effect = 0.12, $se_{Bootstrap}$ = 0.08, 95%CI (0.08, 0.15). (This analysis was run with the PROCESS macro (Hayes, 2013)).

Analyses with age as a continuous variable

Mental health, affective control and emotion regulation capacity across age

There was a significant effect of age on affective control, F (1, 88) = 7.55, p = .007, R^2 = .08, and self-reported emotion regulation difficulties, F (1, 85) = 5.20, p = .025, R^2 = .06. However, the effect of age on mental health difficulties was not statistically significant, F (1, 85) = 2.70, p = .104, R^2 = .03. The effect of age on the experimental measure of emotion regulation was non-significant F (1, 85) = 1.08, p = .300, R^2 = .01.

Affective control and mental health across age

The indirect effect of affective control on the association between age and mental health problems was significant, β = 0.11, *se* = 0.04, *z* = 2.68, *p* = .007, AIC = 748.85.

Figure S1. Emotion regulation and affective control

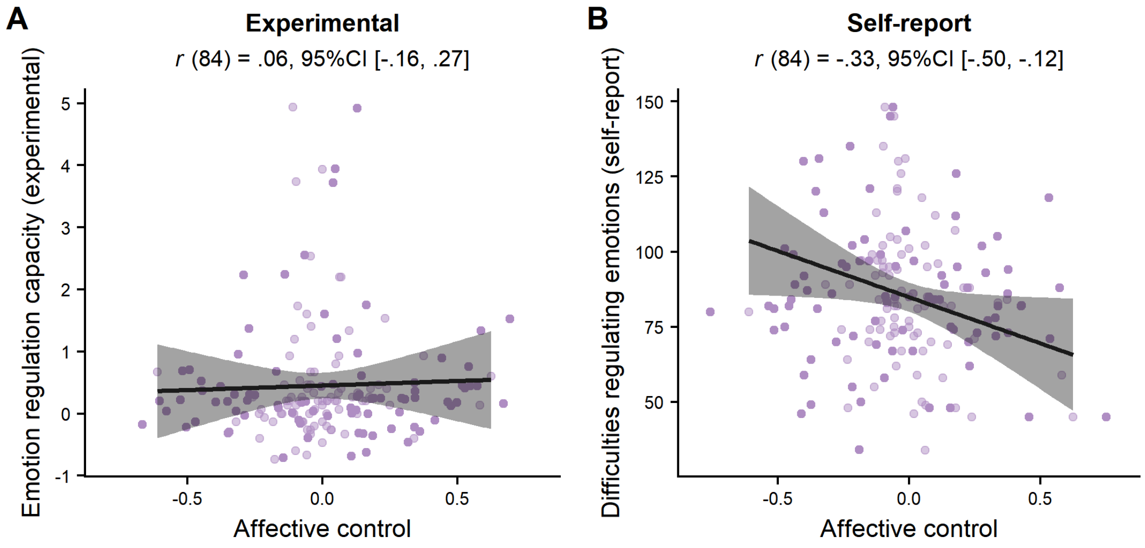

Figure S1. The figure shows the association between affective control emotion regulation assessed experimentally as the difference in distress reported in the Regulate compared to the Negative Watch conditions of the Emotion Regulation Task (**A**) and through self-report measured as the total score on the Difficulties Regulating Emotions Scale (**B**).

Associations between measures of interest

Table S3. Correlations between age, affective control, mental health and emotion regulation

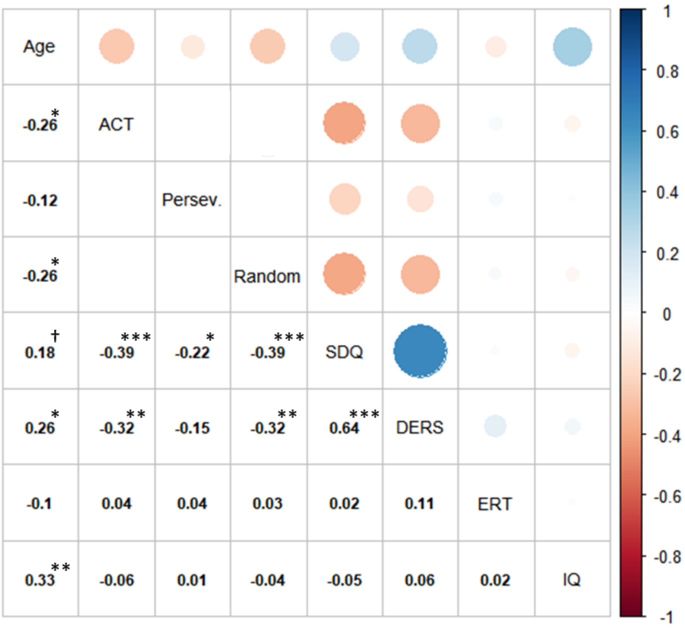

Table S3. Shows the association between all the variables of interest in the current study. † $p \geq .100$, * $p \geq .050$, ** $p \geq .010$, *** $p \geq .001$. ACT = Affective control task, this is the proportional difference score affective relative to neutral non-efficient errors; Persever. = reflects perseverative errors in the affective compared to neutral condition; Random = reflects random errors in the affective compared to neutral condition; SDQ = Strengths and Difficulties Questionnaire (Goodman, 1997), our measure of mental health problems, the total difficulty score on the SDQ; DERS = Difficulties in Emotion Regulation Scale (Gratz & Roemer, 2004), our self-report measure of emotion regulation difficulties, the total score on the DRES; ERT = Emotion Regulation Task, our experimental measure of emotion regulation capacity, cognitive reappraisal capacity, in particular.
